# Wild Ungulate Decision-Making and the Role of Tiny Refuges in Human-Dominated Landscapes

**DOI:** 10.1371/journal.pone.0151748

**Published:** 2016-03-17

**Authors:** Yarlagadda Chaitanya Krishna, Ajith Kumar, Kavita Isvaran

**Affiliations:** 1 Centre for Ecological Sciences, Indian Institute of Science, Bengaluru, Karnataka, India; 2 Manipal University, Manipal, Karnataka, India; 3 Centre for Wildlife Studies, Wildlife Conservation Society – India Program, Bengaluru, Karnataka, India; CSIC-EEZA, SPAIN

## Abstract

Wildlife conservation in human-dominated landscapes requires that we understand how animals, when making habitat-use decisions, obtain diverse and dynamically occurring resources while avoiding risks, induced by both natural predators and anthropogenic threats. Little is known about the underlying processes that enable wild animals to persist in densely populated human-dominated landscapes, particularly in developing countries. In a complex, semi-arid, fragmented, human-dominated agricultural landscape, we analyzed the habitat-use of blackbuck, a large herbivore endemic to the Indian sub-continent. We hypothesized that blackbuck would show flexible habitat-use behaviour and be risk averse when resource quality in the landscape is high, and less sensitive to risk otherwise. Overall, blackbuck appeared to be strongly influenced by human activity and they offset risks by using small protected patches (~3 km^2^) when they could afford to do so. Blackbuck habitat use varied dynamically corresponding with seasonally-changing levels of resources and risks, with protected habitats registering maximum use. The findings show that human activities can strongly influence and perhaps limit ungulate habitat-use and behaviour, but spatial heterogeneity in risk, particularly the presence of refuges, can allow ungulates to persist in landscapes with high human and livestock densities.

## Introduction

Wild animals are known to make remarkably complex decisions when selecting habitats, balancing multiple, often disparate costs and benefits, such as mortality risk against net energy gain, while accounting for diverse ecological factors that influence these costs and benefits, such as resource abundance, predator densities and competition [1–3]. Habitat-selection decisions are rendered even more complex in human-dominated landscapes, where costs and benefits are additionally influenced by human-related factors such as land-use change [4]. Both the ecological and human sets of factors can vary spatially and temporally. Habitat-use decisions in these highly variant ecosystems can have important impacts on the persistence or extinction of a species in a given landscape.

For ungulates, resource availability is influenced by forage quality and quantity, seasonal variation and patchiness in resources [5–7]. Forage quality, in particular, is an important constraint on resource availability since quality decreases with age of the plant and increasing biomass, and varies with phenological status [8,9]. Ungulates respond to resource availability by choosing energy-maximizing strategies at intermediate resource qualities and time-minimizing strategies at high resource qualities [9,10]. Ungulate foraging strategies are also overwhelmingly influenced by predation risk, wherein ungulates minimize the predation risk to foraging rate ratio [11,12].

Ungulate perception of predation risk is greatly influenced by habitat characteristics which determine predator detection, with many ungulates choosing to avoid risky habitats, rather than physical signs, such as presence or odour of predators [3]. As human activities expand into hitherto wild habitats, both resources and risks could be affected directly and indirectly, complicating simplistic ecological hypotheses. Livestock husbandry, an important human activity in many arid and semi-arid ecosystems worldwide, is thought to have an overall negative effect on wild ungulates due to competition for forage or by circumscribing wild ungulates’ habitat-use [13,14]. However, there have also been instances where livestock facilitate wild herbivores by increasing the quantity and quality of forage [15]. By modifying habitats, humans influence habitat characteristics and thus may modify wild herbivores’ perception of risk. There is increasing evidence that wild animals perceive non-lethal human activities also as risks [16]. Increased contact between wild ungulates and humans also occurs when animals feed on agricultural crops occasionally, resulting in human-wildlife conflict [17]. When faced with such a complex and dynamic environment, how do ungulates make habitat-use decisions? Hypotheses about habitat-use decisions in such complex environments have been tested through simulations and under experimental conditions [11,18]. However, how animals balance these diverse and dynamic factors in order to navigate the challenges of co-occurrence with humans in the real world is relatively poorly understood. To our knowledge, no attempt has been made to understand wild ungulate responses when diverse ecological and human-related factors such as changing wild forage quality and quantity, agricultural crop availability, presence of livestock, human activities and risk posed by habitat structure and natural predators occur simultaneously.

We attempt to answer this question in a typical fragmented Indian semi-arid landscape densely populated by humans and livestock where only one large, wild, group-living ungulate, the blackbuck (*Antilope cervicapra*) occurs. This presents an ideal natural environment to study the habitat-use of a wild ungulate without the confounding effects of facilitation and competition by sympatric wild ungulates. Blackbuck are primarily grazers, who prefer open-plains/habitats, as habitat structure represents a form of natural risk [19]. Vigilance in blackbuck is reported to be higher closer to vegetation patches than farther away, suggesting that blackbuck perceive vegetation patches as riskier than open grassland [19]. Blackbuck prefer open grasslands since they rely on early detection and flight to escape their natural predators [19], such as Indian wolves (*Canis lupus pallipes*) which occur in semi-arid landscapes. The open-plains preferred by blackbuck are mostly found in Indian arid and semi-arid zones where resource availability varies sharply through the year in response to precipitation patterns.

To understand how blackbuck habitat-use varies, we hypothesize that blackbuck would show flexible habitat-use behaviour, that is: (1) when overall resource quality in the landscape is high, blackbuck will be risk averse especially with respect to human activities, and, (2) when overall resource quality is low, blackbuck will be less sensitive to risk. Specifically, we predict that: (1) blackbuck would reduce use of riskier unprotected grasslands and increase use of protected habitats when forage quality is high, and, (2) blackbuck would increase use of riskier habitats when forage quality is low.

To test the predictions, multi-year, multi-season blackbuck sign data were collected. Sign data were collected from plots in four habitats (protected grasslands, protected plantations, unprotected grasslands and agricultural areas), which allowed us to examine the influence of diverse ecological and human-related variables on habitat-use in different seasons at fine scales. We used field data to model landscape level habitat-use patterns, and additionally discern the factors influencing habitat-use in protected and unprotected habitats.

## Materials and Methods

### Study area

The Principal Chief Conservator of Forests (Wildlife), Maharashtra State Forest Department granted permission to conduct the study in the Great Indian Bustard Sanctuary in Maharashtra, India.

**Fig 1:**
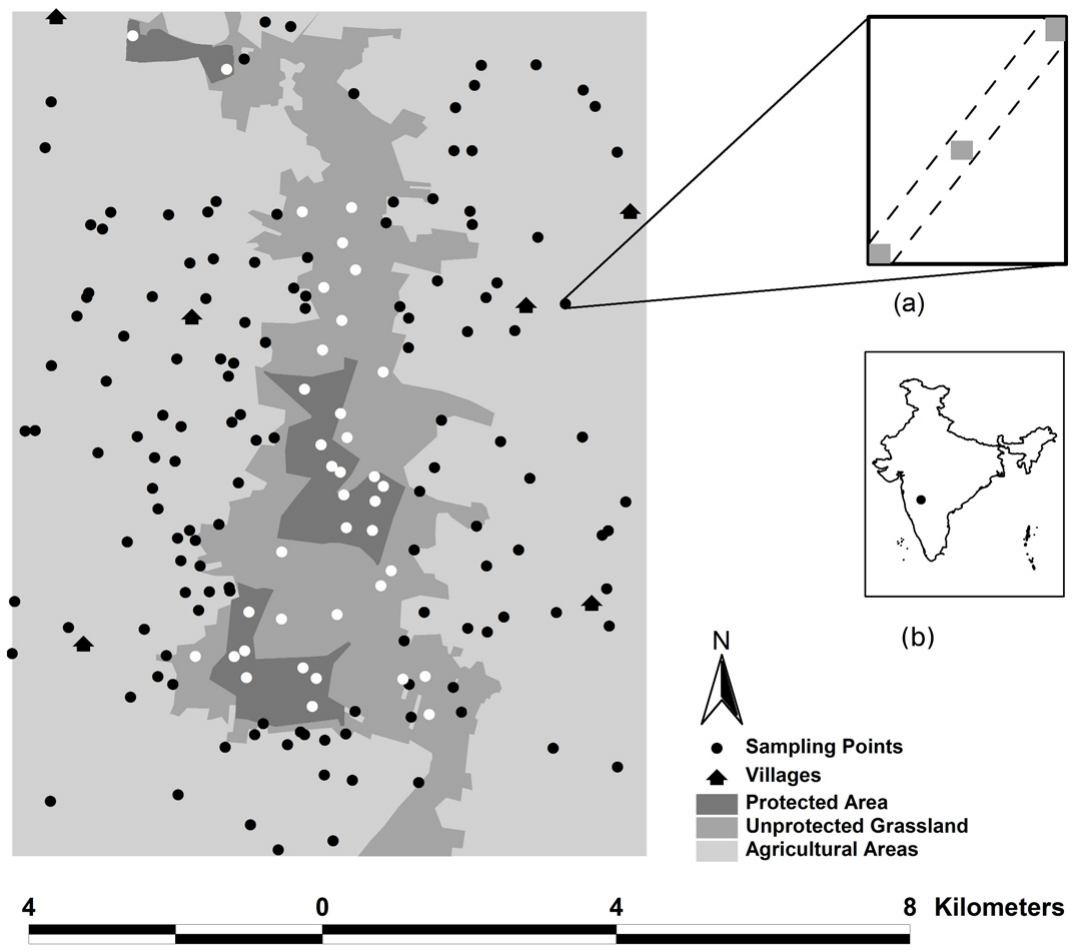
Major habitat types in the study area and blackbuck signs sampling points. Inset figures: (a) The sampling protocol that was followed and (b) Location of the study site in India.

The study was undertaken at the Great Indian Bustard Sanctuary and surrounding areas at Nannaj in India (17°49′N, 75°52′E). The 108 km^2^ study area, with a mean elevation of 505 m above sea level, comprised of protected grasslands and protected plantations or afforested patches, and unprotected grasslands (livestock grazing areas) and agricultural habitats (Fig 1). The human dominated landscape had a population of ~25 000 people in 2001 and a livestock population of ~8 000 bovines (water buffalo (*Bubalus bubalis*) and cattle (*Bos taurus*)), ~7 000 goats (*Capra hircus*) and ~1 400 sheep (*Ovis aries*) in 2007. The annual precipitation is < 750 mm and the semi-arid climate results in a marked seasonality; intense long summers are followed by a brief rainy spell (pre-monsoon) which precedes the full rainy season (monsoon), after which the post-monsoon season leads into the summer. Water is artificially provisioned in the protected area (PA) throughout the year. Blackbuck are known to use available habitats within the landscape and mortality occurs due to poaching (blackbuck are legally protected and hunting blackbuck is banned under Indian wildlife protection laws), traffic (Chaitanya Krishna, personal observations) and predation by Indian wolves (*Canis lupus pallipes*) [20]. At this site, blackbuck off take due to wolves (mean group size = 7.25) was reported to be 4.2–4.6% (25–30 blackbuck) out of the total estimated population of 600–650 blackbuck. Blackbuck constituted 46.5% of all wolf kills and 34% of the kills were within 4 metres from the nearest vegetation cover [20]. Blackbuck poaching attempts were detected on three occasions in the study area. Due to the illegal nature of poaching activities, other poaching attempts might have occurred undetected.

### Landscape level habitat-use patterns

Data on blackbuck habitat-use were collected from November 2008 to August 2010 using indirect signs (tracks and pellets). Thirty eight and 91 sampling points were generated randomly in non-agricultural (protected grasslands, protected plantations and unprotected grasslands) and agricultural habitats respectively, proportional to the area covered by the habitats in the study area (Fig 1). Blackbuck and livestock indirect sign data were collected from a strip transect of dimensions 80x2 m (eight segments of 10 m each), which was centred on each sampling point and oriented in a north-east and south-west direction. The data were collected 7–9 times over the study period at regular intervals to yield 290 data points from non-agricultural habitats and 421 data points from agricultural habitats. The transect length of 80 m was selected to correspond to the diagonal of the average sized field in agricultural habitats, so that data from agricultural and non-agricultural habitats were comparable.

### Resource and risk variables

At each sampling point, habitat openness (visual estimate of percent vegetation > 1 m height in a radius of 5 m) and plant height were measured at both ends and centre of the strip transect (Fig 1a). From a 50x50 cm plot located at the centre of the strip transect, above ground plant (grasses and herbs together) biomass was clipped, weighed and oven dried. Biomass and crop samples were ground and processed using standard laboratory procedures for estimating % Carbon and Nitrogen using a Leco Truspec CN analyzer.

## Data Analysis

### Landscape level habitat-use patterns

Each 10 m segment was given a score of 1 if one or more blackbuck signs were recorded. The individual scores for each 10 m segment were summed for all the eight 10 m segments that constituted the 80x2 m strip transect at each sampling point. The summed score, an index of blackbuck habitat-use could range from 0–8 for each sampling occasion. A similar protocol was followed for livestock signs. The summed signs were compared between protected and unprotected habitats, using means and confidence intervals (CIs).

### Resource and risk variables

Plant height and habitat openness measurements were averaged for each sampling point. The oven-dried grass and herb biomass (plant biomass) and the corresponding carbon to nitrogen (C:N) ratio were used as a measure of forage quantity and quality respectively. The distance of each sampling point to the nearest PA patch boundary was extracted using Google Earth as a surrogate for human activities, which were expected to be unrestricted outside the PA, reduced near the PA boundary and minimal inside the PA. For sampling points inside the PA, distance measurements to the PA boundary were assigned negative values. Due to the strong seasonality at the study site, the nine sampling occasions were categorized into the following seasons (n); summer (4), pre monsoon (1), monsoon (2) and post monsoon (2).

### Modelling landscape level habitat-use

The response variable (blackbuck habitat-use index from indirect signs) was count data, with a large number of zeroes in the dataset and collected repeatedly from sampling points that were spatially fixed. Therefore, generalized linear mixed effects models (GLMMs) in a zero-inflated Poisson (with a log-link) framework were used to examine the effect of ecological and anthropogenic predictors on the response variable. Each sampled occasion at a sampling point (Fig 1) constituted an individual data point. Data from both years were pooled and used in the analysis (n = 290). The predictor variables were checked for multi-collinearity through pair-wise correlations and as plant height and plant biomass were correlated (R = 0.70), plant height was excluded from further analysis.

Each model in the model set included from one to a combination of five variables: plant biomass (forage quantity), C:N ratio (forage quality), distance to PA boundary (human disturbance), livestock and openness (habitat structure—ecological risk). The sampling point was denoted as a random effect. The analysis was run in the software R 2.15.2 [21] using the package ‘glmmADMB’ [22]. An *a priori* candidate set of 27 models which included the null and the global model were formulated based on our hypotheses, our knowledge of the study site, blackbuck behaviour and ecology. Seasonal interactions with all variables except openness were included as we had hypothesized *a priori* that blackbuck would show flexibility in choosing habitats. The global model included seasonal interactions with all variables except openness. Statistical inferences were based on a model selection framework using an information theoretic approach. Model selection was based on corrected Akaike Information Criterion (AICc) and inferences were based on model averaged parameters and 95% CIs calculated using the R package ‘MuMIn’ [23].

To understand the effect of plant biomass on blackbuck, we adopted an additional approach. As blackbuck are known to prefer open habitats and the relationship between plant biomass and blackbuck habitat-use is thus likely to be non-linear, hump-shaped, asymmetric and right-skewed [24], the Ricker function, *Y = aXe*^*-bX*^, was used as it is suitable for modelling such relationships [25]. The relationship was explored via a non-linear least squares approach using the R package ‘nlme’ [26].

### Modelling habitat-use inside and outside the PA

As human activities differed dramatically between protected and unprotected areas (unprotected grasslands), we further explored the factors driving habitat-use by blackbuck in the presence and absence of human-activities by using GLMMs in a zero-inflated Poisson framework separately for the habitats inside (protected grasslands and plantations) and outside (unprotected grasslands) the PA. As very few livestock signs were detected in the PA, the livestock term was dropped from the model set examining habitat-use inside the PA.

## Results

### Landscape level habitat-use patterns

**Fig 2:**
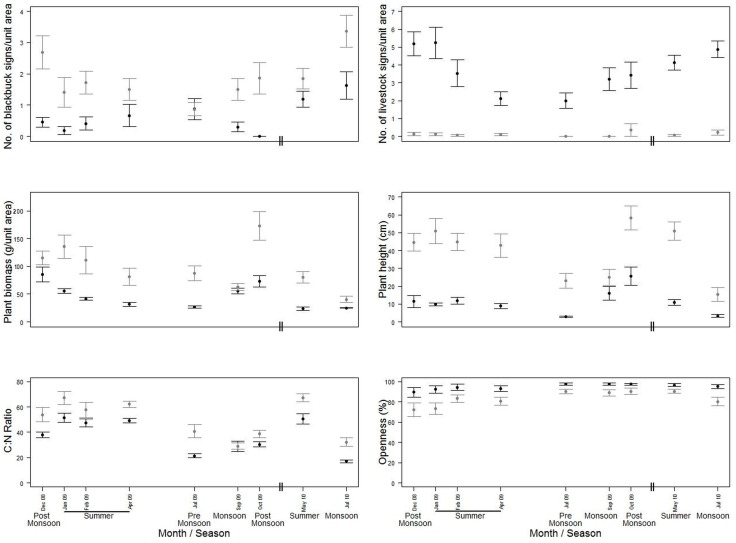
Variation across the study period and between the protected grasslands and protected plantations (grey lines and dots), and, unprotected grasslands (black lines and dots) in; a) blackbuck signs, b) livestock signs, c) plant biomass, d) plant height, e) C:N ratios and f) openness. Lower C:N ratios represents higher quality forage. Means and 95% confidence intervals (bootstrapped) are shown.

Analysis of sign data showed that blackbuck regularly use non-agricultural habitats; signs were detected on 152 occasions out of 290. In contrast, blackbuck signs in agricultural habitats were rare, signs were detected only on 20 occasions out of 421. Due to the scarcity of blackbuck signs in agricultural fields, data from this habitat type were excluded from further analysis. In non-agricultural habitats, blackbuck habitat use was consistently higher in the protected habitats than in the unprotected habitats during the study period (Fig 2).

### Resource and risk factors

The main factors hypothesized to influence blackbuck habitat-use also varied across the year and between the protected grasslands and unprotected grasslands (Fig 2). Forage quantity was always higher in the protected grasslands while forage quality was consistently higher (lower C:N ratios) in the unprotected grasslands. Forage quantity was generally highest in the post-monsoon period and declined towards the summer. Forage quality was generally highest in the monsoon and decreased towards the summer. Livestock signs were higher in the unprotected grasslands with seasonal variation across years.

### Landscape level habitat-use

Models that incorporated seasonal interactions with plant biomass, distance to PA boundary and livestock were the best predictors of habitat-use (Table 1; cumulative Akaike weight of 0.97). Blackbuck habitat-use varied substantially with seasonal interactions on plant biomass (model averaged weight = ~1) and distance (model averaged weight = 0.92) (Table 2). There was only weak support for a relationship between livestock and blackbuck habitat-use modified by season (model averaged weight = 0.42). There was no support for a relationship between habitat-use and C:N ratio or habitat openness (model averaged weights of each variable = 0.03). The strong support for a relationship between habitat-use and; (1) seasonal interactions on plant biomass, and, (2) distance to the PA boundary suggests that blackbuck habitat-use decisions differed seasonally and were hence dynamic.

**Table 1:** Top ranked models from a model set comprising 27 models exploring variables affecting blackbuck signs.

Sl no.	Model	df	logLik	AICc	Delta	Weight
1	Season*Dist + Season*Biomass	14	-338.07	706.00	0.00	0.56
2	Season*Dist + Season*Biomass + Season*Lvs	18	-334.07	707.23	1.22	0.31
3	Season*Biomass + Season*Lvs	14	-340.11	710.08	4.07	0.07
4	Season*Dist + Season*Biomass + Season*Lvs + C:N + Openness	20	-334.07	711.96	5.95	0.03
5	Season*C:N + Season*Dist + Season*Biomass	18	-337.25	713.59	7.58	0.01

Season, four distinct seasons in the study area (Summer, Pre-monsoon, Monsoon and Post-monsoon); Biomass, forage quantity (gm/unit area); Dist, distance (m) to the protected area boundary; Lvs, livestock signs/unit area; Open, habitat openness (%); C:N, forage quality. Model statistics shown are df (degrees of freedom), log-likelihood, Akaike Information Criterion corrected for small sample size, delta AICc and Akaike weights.

**Table 2:** Model averaged β coefficient, 95% confidence intervals and weights associated with habitat use through signs from a model set comprising 27 models.

	B Estimate	95% Lower CI	95% Upper CI	Weights
**Intercept: Season Monsoon**	**1.51**	**0.82**	**2.19**	
**Biomass**	**-0.02**	**-0.03**	**-0.01**	**0.99**
Season				1.00
**Season Post-monsoon**	**-0.91**	**-1.69**	**-0.14**	
**Season Pre-monsoon**	**-1.38**	**-2.74**	**-0.01**	
**Season Summer**	**-1.09**	**-1.81**	**-0.37**	
Season:Biomass				0.99
**Season Post-monsoon:Biomass**	**0.02**	**0.00**	**0.03**	
Season Pre-monsoon:Biomass	0.02	-0.01	0.04	
**Season Summer:Biomass**	**0.02**	**0.01**	**0.03**	
Dist	-0.0006	-0.001	0.0002	0.92
Season:Dist				0.92
**Season Post-monsoon:Dist**	**-0.002**	**-0.003**	**-0.0003**	
Season Pre-monsoon:Dist	-0.0002	-0.002	0.002	
Season Summer:Dist	0.0001	-0.0006	0.0009	
Lvs	-0.08	-0.29	0.12	0.42
Season:Lvs				0.42
Season Post-monsoon:Lvs	-0.09	-0.38	0.20	
Season Pre-monsoon:Lvs	0.56	-0.02	1.14	
Season Summer:Lvs	0.06	-0.16	0.28	
C:N	0.001	-0.02	0.02	0.05
Season:C:N				0.02
Season Post-monsoon:C:N	-0.001	-0.03	0.03	
Season Pre-monsoon:C:N	-0.03	-0.08	0.02	
Season Summer:C:N	-0.004	-0.03	0.02	
Openness	-0.0004	-0.01	0.01	0.03

CI, Confidence Interval; Season, four distinct seasons in the study area (Summer, Pre-monsoon, Monsoon and Post-monsoon); Biomass, forage quantity (gm/unit area); Dist, distance (m) to the protected area boundary; Lvs, livestock signs/unit area; Open, habitat openness (%); C:N, forage quality. Terms in bold indicate 95% confidence intervals that do not include zero.

**Fig 3:**
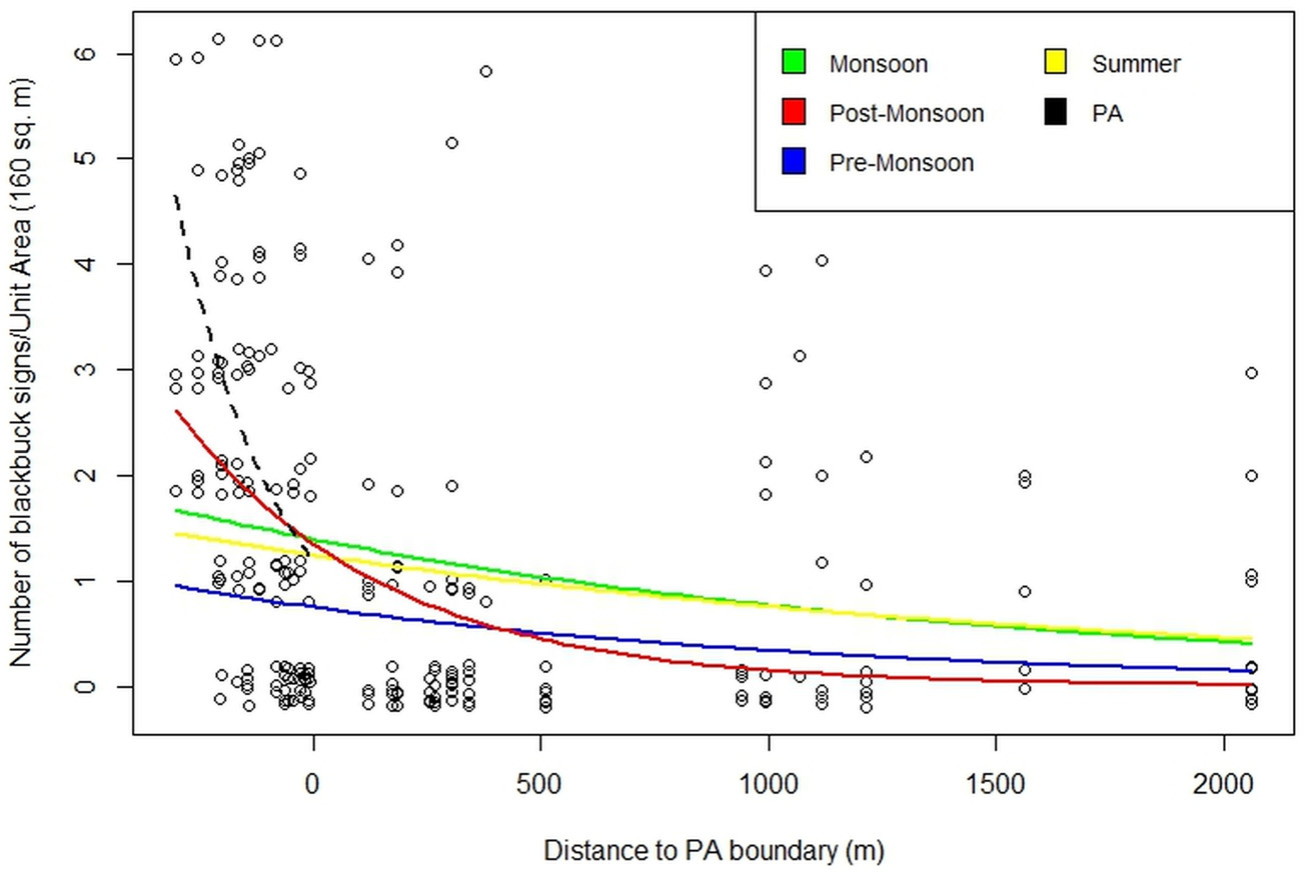
Habitat-use variation in relation to the distance to protected area (PA) boundary. Points to the left of zero on the x-axis are sampling points inside the PA. Lines are GLMM model predictions using model averaged coefficients. Dashed line is the prediction inside the PA.

**Fig 4:**
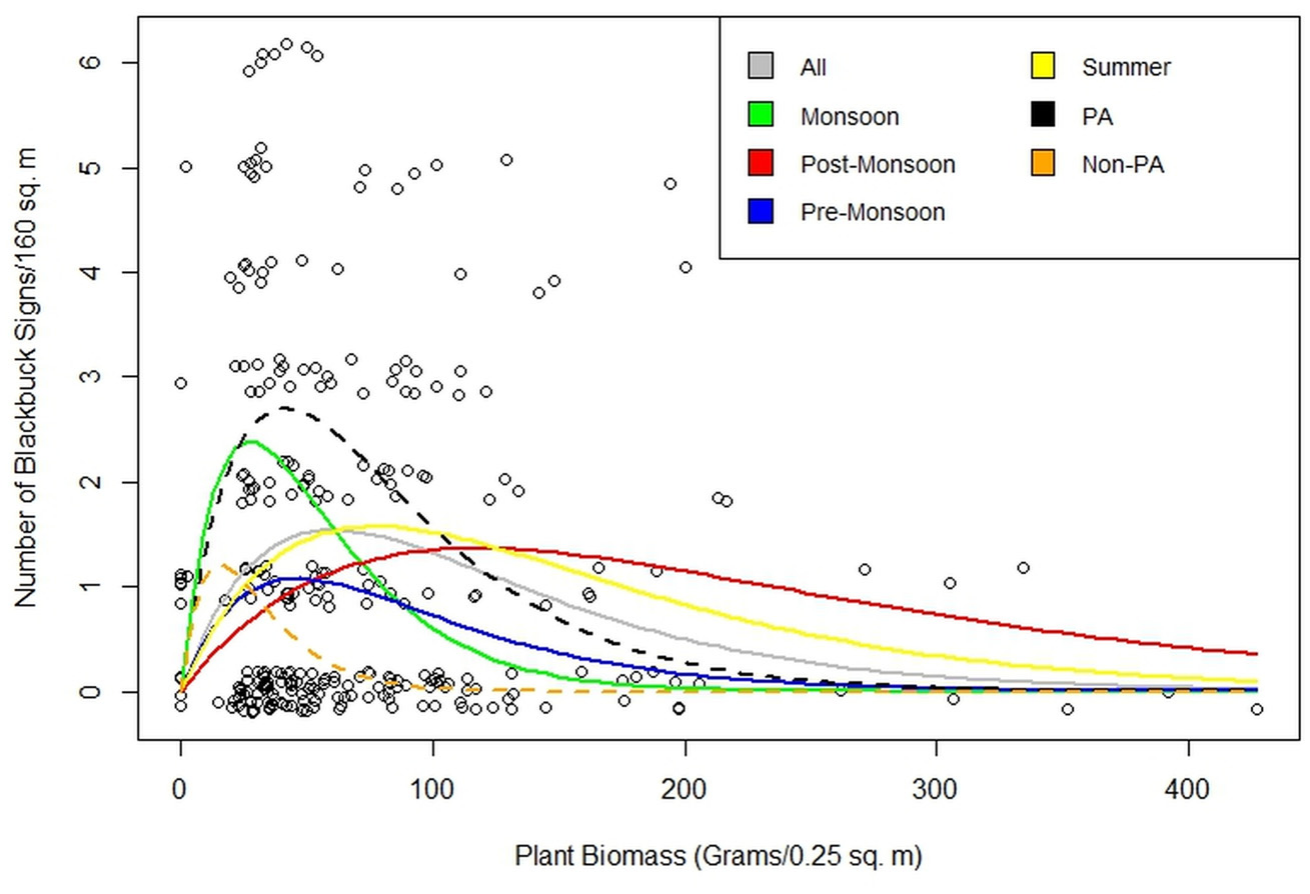
The influence of plant biomass on habitat use, quantified from a non-linear Ricker equation. The four seasons (Post-Monsoon, Summer, Pre-Monsoon and Monsoon) included data from all habitats, the two habitats (PA and Non-PA, shown in dashed lines) included data from all seasons, and, data from all seasons and habitats was included in All.

Habitat-use was negatively related to distance to the PA boundary (from sampling points located inside and outside the PA), a measure of human disturbance, in the post-monsoon season alone based on 95% CIs of the model coefficient (Fig 3; Table 2). Habitat-use was not consistently related to distance to PA boundary in other seasons. Habitat-use was negatively related to forage quantity, represented by plant biomass, in the monsoon and pre-monsoon and this relationship was much weaker in the summer and post-monsoon seasons (Table 2). The influence of plant biomass on habitat-use using Ricker functions (Fig 4), shows that habitat-use clearly peaked at relatively low plant biomass (60.11 g, 95% CI 42–87). When differences across seasons were examined, blackbuck were most selective with respect to plant biomass in the monsoon: the slope of the Ricker function, which describes how steeply the curve declines from the maximum is the steepest (0.24) in the monsoon and blackbuck maximally used areas where the average plant biomass was 27 g (95% CI: 17–44). Blackbuck were less selective in the pre-monsoon and summer: the slope was low (0.06) in the pre-monsoon and summer and the 95% CI indicated that blackbuck used areas across a wide range of plant biomass. Blackbuck were least selective in the post-monsoon: the slope was the lowest (0.03) in the post-monsoon and the 95% CI indicated that blackbuck used areas across a wide range of plant biomass.

### Habitat-use inside the PA (protected grasslands and plantations)

Blackbuck habitat-use was strongly influenced by plant biomass and distance to the PA boundary (S1 and S2 Tables). There was little support for C:N ratio and habitat openness on habitat-use. Blackbuck were very selective with respect to plant biomass in the protected habitats (steep slope of 0.18 in Fig 4), and were primarily using areas where the average above ground biomass was 41 g, (95% CI: 31–55). Interestingly, even within the protected habitats, the distance to PA boundary had a negative effect on habitat-use, revealing that areas close to the PA periphery are being used minimally and core areas are registering maximum use by blackbuck (S1 Table).

### Habitat-use outside the PA (unprotected grasslands)

Habitat-use was overwhelmingly related to plant biomass, which had a negative slope (S3 Table). Blackbuck were very selective with respect to plant biomass in the unprotected habitat, (steep slope of 0.22 in Fig 4), and blackbuck were primarily using areas where the average above ground biomass was 15 g, (95% CI: 9–30). The steeper slope of plant biomass in the unprotected habitat (0.22) compared to its slope in the protected habitat (0.18), suggests that blackbuck were in fact more selective in the unprotected habitat and were on average selecting areas with lower plant biomass (15 g) versus 41 g in the protected habitats. Habitat use was weakly related to C:N ratio. There was no support for relationships between habitat-use and other variables, perhaps because in unprotected habitats, livestock presence was ubiquitous and habitat was open throughout (S3 and S4 Tables).

## Discussion

Our findings support our hypothesis that when overall resource quality in the landscape is high, blackbuck are risk averse especially when making decisions related to human activities, whereas when overall resource quality is low, blackbuck are less sensitive to risk. We find a strong influence of human activity on habitat-use by blackbuck and also a role for small protected refuges in facilitating habitat-use in a heavily human-dominated landscape. In the study area, a fragmented human and livestock-dominated agricultural landscape, blackbuck are faced with two seasonally changing scenarios; small protected habitat patches having high quantity but low quality forage with minimal risk (livestock and human activities), and unprotected habitats much larger in extent having low quantity but high quality forage with considerable risk (livestock and human activities). Overall, blackbuck appear to strongly avoid the habitat with higher risk (human-related): habitat-use was very strongly associated with habitat protection; blackbuck signs were consistently higher in protected habitats than in unprotected habitats.

Our findings contrast with studies on other wild ungulates that report an important role played by forage quality [27,28]. As relatively small-bodied ruminants (30–40 kg) that largely specialise on grass, a type of forage that is of relatively low-quality for large parts of the year, forage quality is strongly expected to influence blackbuck habitat-use [28]. Forage quality surprisingly did not seem to be an important ecological condition influencing habitat-use, perhaps because of the risk associated with the areas of high forage quality (unprotected grasslands). Instead, forage quantity emerged as an important predictor of habitat-use, but there was dramatic seasonal variation. In the summer, habitat-use was largely associated with forage quantity with risk not playing a significant role, while in the post-monsoon season, it was associated with both forage quantity and risk, with blackbuck minimizing risk by largely using protected habitats. As the post-monsoon season is the peak plant growing period in the study area, adequate forage was likely available even in small patches of protected habitat. Simultaneously, an increase in the presence of livestock in the unprotected habitat during this period would have represented an increased predation risk. It is likely that the human herders and dogs which accompany livestock are responsible for the perceived increase in predation risk by blackbuck in this landscape, similar to ungulates elsewhere [14]. These results seem to suggest that when blackbuck can afford to avoid risk when resources are abundant, they do so. Seasonal variation was also observed in the selectivity of forage quantity. While generally blackbuck habitat use peaked at intermediate biomass, blackbuck were most selective of forage quantity in the monsoon, where they chose areas of low biomass. This pattern possibly represents avoidance of low-visibility (tall-grass) patches and/or selection of more palatable forage, since digestibility often decreases as grasses mature and increase in height [24]. The degree of forage quantity selectivity was greater in the unprotected habitat than in the protected habitats, implying that blackbuck were being very selective in unprotected habitats to perhaps minimize time spent in riskier habitats. In summary, we find that blackbuck respond dynamically to seasonally-changing levels of resources and risks, with the protected habitats registering maximum use.

Most other studies examining the role of forage characteristics are from areas with low human presence. However, a similar pattern of risk dominating resources in influencing animal habitat-use decisions has been reported by studies of systems with natural predators [29,30]. There is some evidence, again with natural predators, that when forage distribution is severely limited, ungulate response to risk diminishes [31]. Like our study, several other studies of wild ungulates report a relationship between habitat-use and forage quantity [10,27,28]. Some also report a peak in use at intermediate biomass [10,27,32].

Ecological understanding of ungulate decision making, including of the influence of human activities, has largely been from studies in large wildlife reserves, for e.g., Yellowstone National Park [33]. While it is well established that wild animals perceive even non-lethal human activities as risks [16], studies that have investigated the effect of human activities on ungulates have largely focussed on relatively low-intensity activities. For e.g., in Yellowstone, in areas with brown bears, pregnant moose moved closer to roads to give birth as brown bears are road-averse, while this pattern was not observed either in non-pregnant female moose or in brown bear free areas [34]. Our findings suggest that human activities can strongly influence and perhaps limit ungulate habitat-use and behaviour, but spatial heterogeneity in risk, particularly the presence of refuges, can allow ungulates to persist in landscapes with high human and livestock densities. The use of refugia by prey species, in response to natural predators [33], suggests that irrespective of the source of the risk, be it natural or anthropogenic, animals avoid risk by using refuges available within a landscape. Two small PA patches (~3 km^2^ each) serve as refugia for blackbuck in a fragmented agricultural-grassland mosaic. These tiny refuges have enabled the blackbuck population of 250–300 animals to persist in this landscape from the 1980s till date ([17,20], this study).

Human activities are expanding evermore into previously undisturbed habitats with attendant negative effects on large wild ungulates. In this changing scenario, it is important to identify the range and quantum of human-wildlife interactions which will allow large wild ungulates to persist while safeguarding human economic interests. In developing countries densely populated with people and livestock, it is critical that balanced approaches are identified to meet both biodiversity conservation and social goals. We find that even small well-protected areas are able to buffer a large wild ungulate against the risks posed by human activities. While small PAs cannot buffer wildlife against disease transmission from livestock, climate change and are inadequate for migratory ungulates, our study shows that they play an important role in the persistence of certain types of wild ungulates in a human-dominated landscape. These results are important as they show that it is possible to find mechanisms through which wildlife and human interests can perhaps be met simultaneously.

## Supporting Information

S1 Table Top ranked models from a model set comprising 17 models exploring variables affecting blackbuck habitat use in protected grasslands and plantations. (PDF)

S2 Table Model averaged β co-efficients, 95% confidence intervals for variables and model-averaged weights for variables affecting blackbuck habitat use in protected grasslands and plantations from a model set comprising of 17 models. (PDF)

S3 Table Top ranked models from a model set comprising 27 models exploring variables affecting blackbuck habitat use in unprotected grasslands. (PDF)

S4 Table Model averaged β co-efficients, 95% confidence intervals for variables and model-averaged weights for variables affecting blackbuck habitat use in unprotected grasslands from a model set comprising of 27 models. (PDF)
