# Supplementary material for: Wild Ungulate Decision-Making and the Role of Tiny Refuges in Human-Dominated Landscapes
Source: PLoS One. 2016 Mar 17;11(3):e0151748. doi: 10.1371/journal.pone.0151748 (PMC4795686; doi:10.1371/journal.pone.0151748)
Supplement: S1 Table — (PDF) [file pone.0151748.s001.pdf]

S1 Table. Top ranked models from a model set comprising 17 models exploring variables affecting blackbuck habitat use in protected grasslands and plantations.

Sl no.	Model	df	logLik	AICc	Delta	Weight
1	Season*Dist + Season*Biomass	14	-203.18	438.12	0.00	0.49
2	Season*Biomass	10	-208.49	438.88	0.76	0.33
3	C:N + Dist + Biomass + Openness	7	-213.20	441.34	3.22	0.10
4	Openness + Season*Dist + Season*Biomass	16	-203.10	443.16	5.04	0.04
5	Biomass	4	-218.32	444.97	6.85	0.02
6	Season*Dist	10	-211.69	445.27	7.16	0.01
7	C:N + Biomass	5	-218.29	447.08	8.96	0.01

Season, four distinct seasons in the study area (Summer, Pre-monsoon, Monsoon and Post-monsoon); Biomass, forage quantity (gm/unit area); Dist, distance (m) to the protected area boundary; Open, habitat openness (%); C:N, forage quality.

Model statistics shown are df (degrees of freedom), log-likelihood, Akaike Information Criterion corrected for small sample size, delta AICc and Akaike weights.
