# Supplementary material for: Wild Ungulate Decision-Making and the Role of Tiny Refuges in Human-Dominated Landscapes
Source: PLoS One. 2016 Mar 17;11(3):e0151748. doi: 10.1371/journal.pone.0151748 (PMC4795686; doi:10.1371/journal.pone.0151748)
Supplement: S2 Table — (PDF) [file pone.0151748.s002.pdf]

S2 Table. Model averaged β co-efficients, 95% confidence intervals and weights for variables affecting blackbuck habitat use in protected grasslands and plantations from a model set comprising of 17 models.

	**β Estimate**	**95% Confidence Intervals**		**Weights**
		**Lower**	**Upper**	
**Intercept: Season Monsoon**	**1.1827**	**0.0507**	**2.3147**	
**Biomass**	**-0.0165**	**-0.0310**	**-0.0020**	**0.98**
Season				0.88
Season Post-monsoon	-0.4832	-1.5410	0.5746	
Season Pre-monsoon	-1.5852	-3.4486	0.2782	
Season Summer	-0.6367	-1.6056	0.3321	
Season:Biomass				0.87
Season Post-monsoon:Biomass	0.0124	-0.0012	0.0259	
Season Pre-monsoon:Biomass	0.0147	-0.0054	0.0348	
Season Summer:Biomass	0.0135	-0.0002	0.0273	
**Dist**	**-0.0045**	**-0.0080**	**-0.0010**	**0.64**
Season:Dist				0.54
Season Post-monsoon:Dist	-0.0010	-0.0058	0.0038	
Season Pre-monsoon:Dist	-0.0003	-0.0085	0.0080	
Season Summer:Dist	0.0015	-0.0024	0.0055	
Openness	-0.0013	-0.0120	0.0095	0.14
C:N	-0.0005	-0.0111	0.0100	0.15
Season:C:N				0.01

Season Post-monsoon:C:N	-0.0089	-0.0380	0.0201	
Season Pre-monsoon:C:N	-0.0214	-0.0771	0.0342	
Season Summer:C:N	-0.0099	-0.0388	0.0190	

Season, four distinct seasons in the study area (Summer, Pre-monsoon, Monsoon and Post-monsoon); Biomass, forage quantity (gm/unit area); Dist, distance (m) to the protected area boundary; Open, habitat openness (%); C:N, forage quality.

Terms in bold indicate 95% confidence intervals that do not overlap zero.
