# Supplementary material for: Wild Ungulate Decision-Making and the Role of Tiny Refuges in Human-Dominated Landscapes
Source: PLoS One. 2016 Mar 17;11(3):e0151748. doi: 10.1371/journal.pone.0151748 (PMC4795686; doi:10.1371/journal.pone.0151748)
Supplement: S3 Table — (PDF) [file pone.0151748.s003.pdf]

S3 Table. Top ranked models from a model set comprising 27 models exploring variables affecting blackbuck habitat use in unprotected grasslands.

Sl no.	Model	df	logLik	AICc	Delta	Weight
1	Biomass	4	-125.21	258.78	0.00	0.58
2	C:N + Biomass	5	-125.01	260.58	1.80	0.23
3	Season*Biomass	10	-119.95	262.04	3.26	0.11
4	Season*C:N	10	-121.31	264.76	5.98	0.03
5	Season*Lvs	10	-122.09	266.32	7.54	0.01
6	C:N + Biomass + Dist + Openness + Lvs	8	-124.71	266.79	8.01	0.01
7	Season*Dist	10	-122.56	267.25	8.47	0.01

Season, four distinct seasons in the study area (Summer, Pre-monsoon, Monsoon and Post-monsoon); Biomass, forage quantity (gm/unit area); Dist, distance (m) to the protected area boundary; Lvs, livestock signs/unit area; Open, habitat openness (%); C:N, forage quality.

Model statistics shown are df (degress of freedom), log-likelihood, Akaike Information Criterion corrected for small sample size, delta AICc and Akaike weights.
