# Supplementary material for: Wild Ungulate Decision-Making and the Role of Tiny Refuges in Human-Dominated Landscapes
Source: PLoS One. 2016 Mar 17;11(3):e0151748. doi: 10.1371/journal.pone.0151748 (PMC4795686; doi:10.1371/journal.pone.0151748)
Supplement: S4 Table — (PDF) [file pone.0151748.s004.pdf]

S4 Table. Model averaged β co-efficients, 95% confidence intervals and weights for variables affecting blackbuck habitat use in unprotected grasslands from a model set comprising of 27 models.

	**β Estimate**	**95% Confidence Intervals**		**Weights**
		**Lower**	**Upper**	
Intercept: Season Monsoon	1.17200	-0.037	2.381	
**Biomass**	**-0.03256**	**-0.057**	**-0.008**	**0.94**
C:N	-0.00899	-0.045	0.027	0.28
Season				0.17
Season Postmonsoon	-2.42900	-5.145	0.286	
Season Premonsoon	-0.35010	-5.085	4.385	
Season Summer	-1.59200	-4.246	1.063	
Season:Biomass				0.12
Season Postmonsoon:Biomass	0.03047	-0.022	0.083	
Season Premonsoon:Biomass	0.01661	-0.173	0.206	
Season Summer:Biomass	0.05986	-0.005	0.125	
Season:C:N				0.03
Season Postmonsoon:C:N	0.05453	-0.039	0.148	
Season Premonsoon:C:N	-0.03617	-0.179	0.107	
Season Summer:C:N	0.03812	-0.042	0.118	
Lvs	0.05981	-0.153	0.272	0.03
Season:Lvs				0.02
Season Postmonsoon:Lvs	-0.08811	-0.459	0.282	

Season Premonsoon:Lvs	0.06754	-0.380	0.516	
Season Summer:Lvs	-0.08526	-0.406	0.235	
Dist	0.00003	-0.001	0.001	0.02
Season:Dist				0.01
Season Postmonsoon:Dist	-0.00040	-0.002	0.001	
Season Premonsoon:Dist	-0.00043	-0.002	0.001	
Season Summer:Dist	-0.00006	-0.001	0.001	
Openness	0.01151	-0.028	0.051	0.01

Season, four distinct seasons in the study area (Summer, Pre-monsoon, Monsoon and Post-monsoon); Biomass, forage quantity (gm/unit area); Dist, distance (m) to the protected area boundary; Lvs, livestock signs/unit area; Open, habitat openness (%); C:N, forage quality.

Terms in bold indicate 95% confidence intervals that do not overlap zero.
